# Engineered optical properties of silver-aluminum alloy nanoparticles embedded in SiON matrix for maximizing light confinement in plasmonic silicon solar cells

**DOI:** 10.1038/s41598-017-12826-1

**Published:** 2017-10-02

**Authors:** Piyush K. Parashar, Vamsi K. Komarala

**Affiliations:** Centre for Energy Studies, Indian Institute of Technology Delhi, New Delhi, 110016 India

## Abstract

Self-assembled silver-aluminum (Ag-Al) alloy nanoparticles (NPs) embedded in SiO_2_, Si_3_N_4,_ and SiON dielectric thin film matrices explored as a hybrid plasmonic structure for silicon solar cells to maximize light confinement. The Ag_2_Al NPs prepared by ex-vacuo solid-state dewetting, and alloy formation confirmed by X-ray diffraction and photoelectron spectroscopy analysis. Nanoindentation by atomic force microscopy revealed better surface adhesion of alloy NPs on silicon surface than Ag NPs due to the Al presence. The SiON spacer layer/Ag_2_Al NPs reduced silicon average reflectance from 22.7% to 9.2% due to surface plasmonic and antireflection effects. The SiON capping layer on NPs reduced silicon reflectance from 9.2% to 3.6% in wavelength region 300–1150 nm with preferential forward light scattering due to uniform Coulombic restoring force on NPs’ surface. Minimum reflectance and parasitic absorptance from 35 nm SiON/Ag_2_Al NPs/25 nm SiON structure reflected in plasmonic cell’s photocurrent enhancement from 26.27 mA/cm^2^ (of bare cell) to 34.61 mA/cm^2^ due to the better photon management. Quantum efficiency analysis also showed photocurrent enhancement of cell in surface plasmon resonance and off-resonance regions of NPs. We also quantified dielectric thin film antireflection and alloy NPs plasmonic effects separately in cell photocurrent enhancement apart from hybrid plasmonic structure role.

## Introduction

The photon management in silicon (Si) solar cells is never ending quest, emerging nanophotonics/plasmonics ideas are also providing an alternate option for the light confinement/trapping in Si wafers apart from conventional light randomization techniques. For localized surface plasmon resonances (LSPRs) assisted light trapping (resonant light path enhancement) in Si solar cells, the metal nanostructures have been the choice^1–5^. The selection of metal for plasmonics application depends on the dielectric constant (density of conduction band electrons plays a role) and interband transitions^3,5–7^. For plasmonic silicon solar cells (PSSCs); the very common metal has been silver (Ag) due to favourable dielectric properties and material stability^2–4^. But, the Ag nanoparticles (NPs) exhibited a reduction in photocurrent of PSSCs near the LSPR region due to the Fano resonance^8^, and parasitic absorption losses after integrating on the front side of a silicon cell^2,3^, and also the Ag NPs possess poor surface adhesion^9^.

As an alternative low cost and abundant material, aluminum (Al) NPs are investigated as broadband plasmonic light scatterers for Si solar cells due to the weaker interband damping of dipolar LSPRs despite the difficulty in NPs formation^10,11^. The Al also acts like Drude metal below and above the interband transition region, and also has the longer LSPRs dephasing time^6,12^. The Al NPs support LSPRs in blue and UV regions of the light spectrum, which can diminish the possibility of the Fano resonance losses^10,13^. In addition, the Al also exhibits large surface adhesion to a supporting substrate due to the larger shear stress^9^, which may lead to undesired high surface coverage of NPs. However, surface oxidation of the Al NPs is a major issue; oxidized surface (shell layer) can also be exploited for better light forward scattering into the Si solar cells due to modification in the dielectric environment^10^.

Metal nanocomposites like; core-shell and bimetallic/alloys possess unique physico-chemical properties (after tuning dielectric function) with different composition of metal at the nanoscale than their constituent metals. This approach can open up a broad range of applications in nanophotonics, biomedical, and also for energy harvesting^14–18^. For broad wavelength light forward scattering into a Si wafer and to avoid undesirable surface oxidation of the NPs, a dielectric matrix of lower refractive index medium than a Si was used for embedding the NPs (Si/spacer layer/metal NPs/capping layer)^4,19,20^. A thin dielectric spacer layer between the metal NPs and high index substrate can modulate the metal NP’s near-field incoupling into a substrate due to graded refractive index medium^19^, and also can help in reducing electronic trap states at the front surface of Si wafer to minimize the surface recombination^21^. Whereas a capping layer on metal NPs prevents surface oxidation^4^, and then the Fano resonance loss by influencing the polarization charges at the NP’s surface^19,22^.

The SiO_2_ and Si_3_N_4_ are commonly adopted dielectric materials as spacer/capping layers for tuning the LSPRs’ fields of the metal NPs^5,19^. However; silicon oxynitride (SiON) film has the lower parasitic absorption losses, tunable refractive index medium^23^, low density of surface states, and has large energy band gap (5–9 eV); which makes the SiON film optically and electrically more promising than the conventional SiO_2_ and Si_3_N_4_ dielectric films^24^. Still, there is a scope for improving the light absorption in a weakly absorbing Si wafer by engineering front anti-reflection layer with the plasmonics concept by minimization of parasitic absorption, surface oxidation, and the Fano resonance losses. In this work, we try to investigate and exploit the positive effects of a hybrid plasmonic structure consisting of alloy metal NPs and complex dielectric layer for better photon management in silicon solar cells using the simple sputter deposition technique.

## Results and Discussions

### Structural Properties

**Figure 1:**
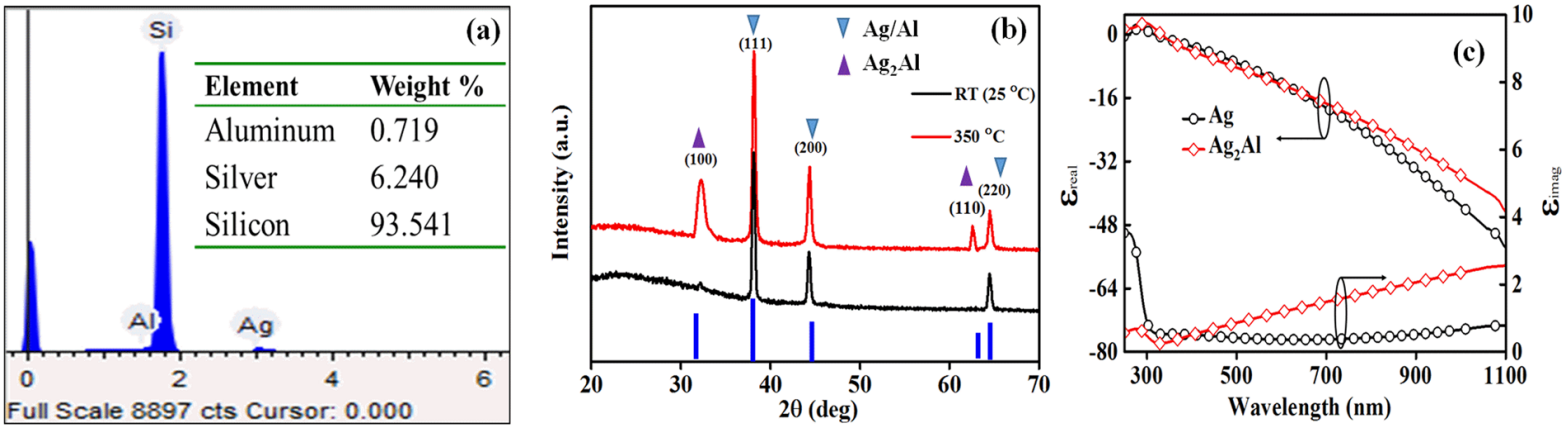
(**a**) EDX of the Ag-Al NPs, (**b**) XRD patterns of the Ag-Al NPs before and after annealing at 350 °C, and (**c**) Experimentally measured real and imaginary dielectric components of pure Ag and Ag-Al NPs.

Figure 1a shows the elemental composition of the Ag-Al NPs on a Si wafer, inset of figure shows detected elements weight percentage; the Al is 10.34 ± 1.20 wt. % of total metal composition. Figure 1b shows the XRD patterns of as-deposited Ag-10 wt. % Al thin film, and in the NPs form after annealing at 350 °C for 1 hour; the standard Al/Ag planes are also presented for reference. The diffraction peaks of Ag/Al at 38.17°, 44.34° and 64.45° are observed, which correspond to (111), (200) and (220) planes, respectively. A small diffraction peak also observed at ~32.20° from the as-deposited film, which is related to (100) plane of an intermetallic compound of the Ag_2_Al (δ-phase)^17^. After annealing, the intensity of the (100) peak enhanced, and an additional peak at ~62.42° related to the (110) plane also observed, which is evidence for the formation of δ-phase of Ag_2_Al alloy NPs^18^. The pure Ag/Al and δ-phase of Ag_2_Al have shown the intense (111) and (100) peaks, respectively, which is an indication of the good quality crystalline lattice in each of NPs grains. The Ag/Al crystal lattice parameter of (111) plane is 0.4082 nm, which is nearly same (0.4089 nm) as reported in literature^15^, whereas the Ag_2_Al crystal lattice parameter of (100) plane is 0.4811 nm.

Figure 1c signifies alteration in real and imaginary dielectric constants of the Ag_2_Al alloy NPs than the pure Ag NPs; the graphs are obtained after fitting the ellipsometry data using Gen-Osc model. The ε_real_ of Ag_2_Al NPs has exhibited a small change at longer wavelength region, but a significant change in the ε_imag_ after 500 nm due to the interband transition (IT) region of Al^6^, in comparison to the Ag NPs. The IT region of the Ag is around 320 nm (between the d-like and sp-like bands near the *L* symmetry axis), whereas for the Al active IT region is ~820 nm (near the *W* point transition between a pair of parallel bands around the Σ axis on the Γ-K-W-X plane)^12^. The Al does not have any d-band for contributing to the valence band, so, the distortion in the Ag lattice occurs with the Al replacement, which can appear in the shift of L_2_ → L_1_ transitions^25^. The shift in L_2_ → L_1_ transition after the alloy formation can tailor the IT and LSPR positions in comparison to the pure Ag NPs. Thus, the alloying of Ag with Al can help to reduce the parasitic absorptance by the alloy NPs than the pure Ag NPs^3,26^.

**Figure 2:**
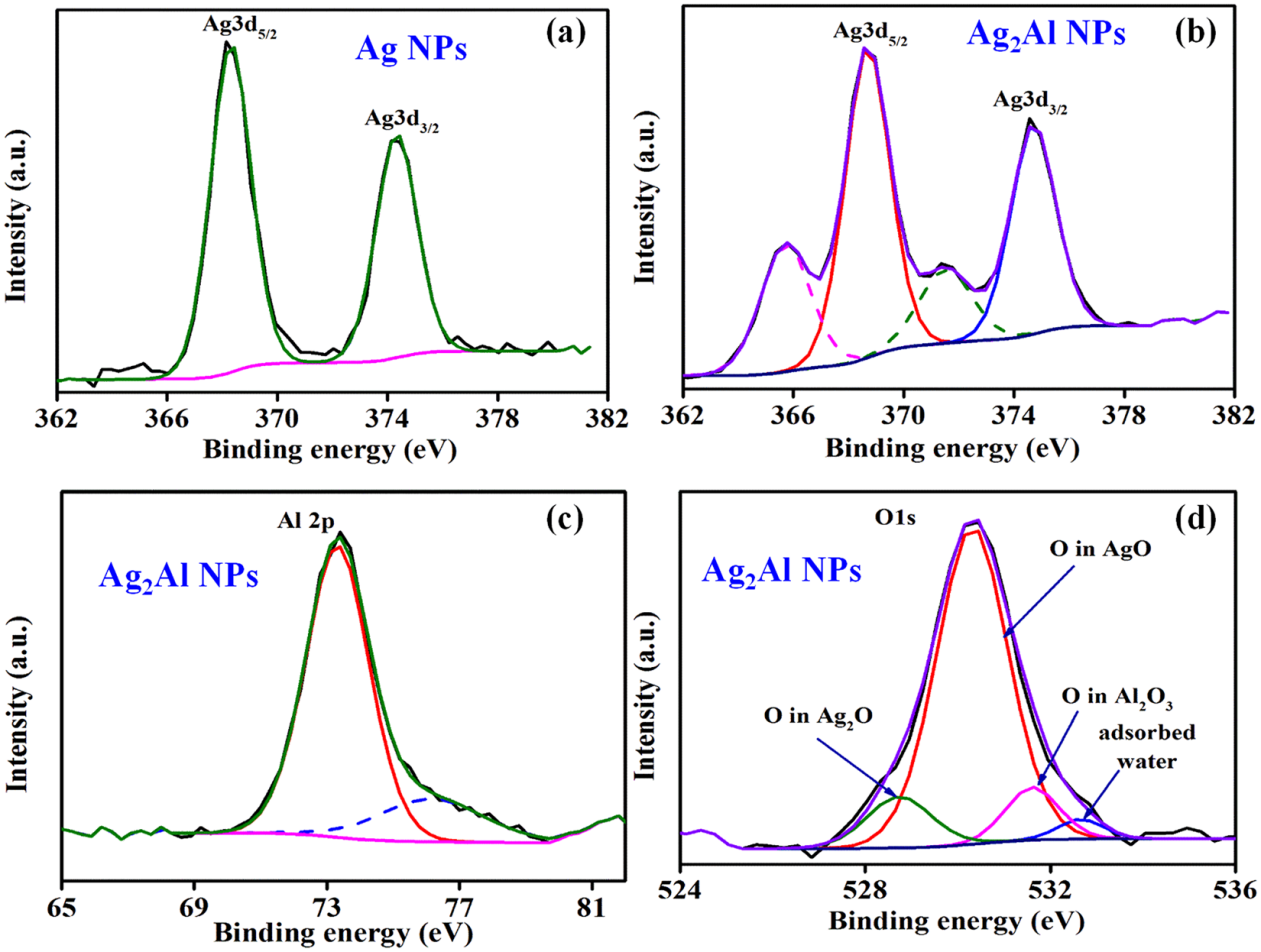
XPS spectra of the Ag NPs’ (**a**) Ag 3d energy level, and the Ag_2_Al alloy NPs’ (**b**) Ag 3d, (**c**) Al 2p and (**d**) O 1 s energy levels.

The δ-phase of Ag_2_Al NPs is further explored by a complementary technique like XPS, which can provide essential information relating to the chemical bonding and oxidation states of the elements. Figure 2 shows core energy levels of the pure Ag NPs’ Ag 3d state, and the Ag_2_Al alloy NPs’ Ag 3d, Al 2p, O 1 s states. The Ag 3d spectrum has shown two peaks related to the Ag 3d_5/2_ and Ag 3d_3/2_ core energy levels (due to the spin-orbit coupling), whose binding energies (B. E.) are 368.2 ± 0.1 eV and 374.2 ± 0.1 eV, respectively (Fig. 2a). The B. E. values are in good agreement with the reported Ag NPs^16^, as well as with the bulk Ag^27^. The Ag_2_Al NPs’ Ag 3d_5/2_ and 3d_3/2_ core energy levels are 368.7 ± 0.1 eV and 374.7 ± 0.1 eV, respectively, which are slightly larger (+0.5 eV) than the pure Ag NPs (Fig. 2a). The shoulders are also observed at lower energy side, which suggests the existence of Ag^+^ oxidic state (Ag_2_O)^16,27^. The Al 2p spectrum (Fig. 2c) has shown two peaks; one at 73.4 ± 0.1 eV related to metallic Al 2p (Al^0^) core level, and another one at 75.9 ± 0.1 eV related to the Al^3+^ state of Al_2_O_3_ phase^28^. In O 1 s spectrum (Fig. 2d), we have observed three peaks at 528.7 ± 0.1 eV, 531.8 ± 0.1 eV and 530.2 ± 0.1 eV energy positions, which are of oxygen in Ag_2_O, AgO, and Al_2_O_3_ phases, respectively, a small peak ~532.3 ± 0.1 eV relating to the adsorbed water is also observed^29^.

Our main observation from the XPS data is; the core energy levels of Ag (Fig. 2a) appeared slightly at the higher binding energy than those reported in the literature^25^. This variation can be attributed to difficulties in interpreting X-ray emission from the metals, where the ‘d’ energy bands lie a few electronvolts just below the Fermi energy level. The core energy level peaks exhibited a shift to the higher energy side upon alloying the Ag NPs (Fig. 2b), which indicates the decrease of electron density at an atomic site in comparison to simple ionic compounds^16,28^. This B. E. shift can also the partial coverage of surfaces by chemisorbed species or oxides that can change the electronic environment around the Ag atoms. Also, the narrower photoemission spectra of the alloy NPs’ d-bands (of the Ag) are observed in comparison to the pure Ag (Fig. 2b). With the Al presence in Ag matrix, the reactivity with oxygen is observed (Fig. 2b–d), but does not appear in the XRD pattern (Fig. 1b). Yang *et al*. only observed the Al_2_O_3_ signature in the XRD spectrum after >20 wt. % of Al in the Al-Ag alloy thin films^17^.

**Figure 3:**
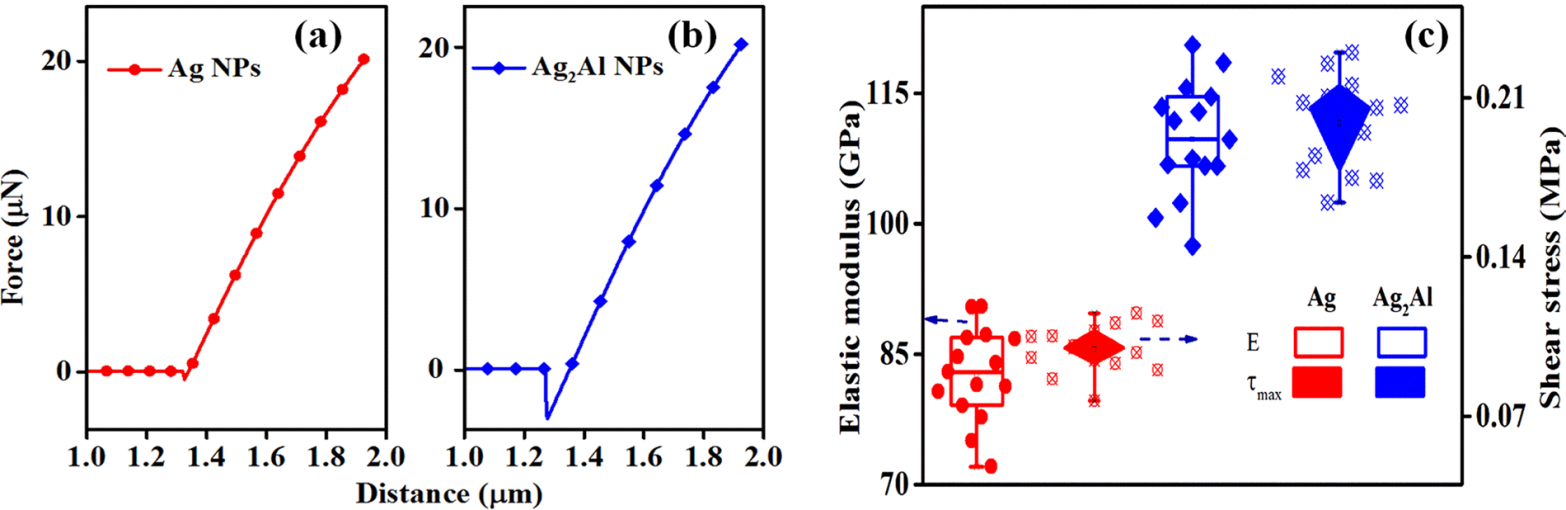
(**a**) Force versus distance curves retrieved by the nanoindentation for (**a**) Ag NPs and (**b**) Ag_2_Al NPs; and (**c**) Elastic modulus and maximum shear stress of the Ag and Ag_2_Al NPs.

It is essential to understand how the NPs adhere to a substrate for integrating on a device structure. The cohesion between the substrate and discontinuous metal film will affect the NPs’ morphology (size, shape, and coverage) while minimizing the energy of the system during the NPs preparation (dewetting process). The NPs’ adhesion on a Si substrate can be analyzed by the shear stress (τ_max_) estimation (it is a function of Young’s modulus E), which we have obtained from the force curves (based on sample-tip interactions) after the AFM indentation. Figure 3a and b show the force versus distance (piezo ‘z’ position) curves for the Ag and Ag_2_Al NPs, respectively. Here, the Si tip (Poisson ratio = 0.3 and spring constant = 42 N-m^−2^) is used for indentation instead of the diamond coated tip (for hardness measurements). Retraced force curves are analyzed by the Nanoscope software (Derjanguin-Muller-Toporov fit model) for the NPs’ Young modulus estimation; the fit model assumes the contact profile remains the same as in the Hertzian model with inclusion of an adhesion force^30^. From the Fig. 3b, one can observe the larger downward piezo deflection for the alloy Ag_2_Al NPs than the pure Ag NPs, which revealed the more adhesive force between the tip and Ag_2_Al NPs, which is an indication of the better adhesion of the alloy NPs to a Si surface.

To support the above statement, we also estimated τ_max_ values using following relation and presented in the Fig. 3c. The τ_max_ of the system can be written in an elastic regime as^31^;

1 $${\tau }_{max}=\frac{0.31}{\pi }{[6{P}_{max}(\frac{{E}_{s}}{1-{\vartheta }_{s}^{2}})]}^{1/3}$$

where the P_max_ is peak load applied to a cantilever, the E_s_ and ν_s_ are Young’s modulus and Poisson’s ratio of the sample, respectively. The Poisson’s ratio of the Ag and Al alloy are 0.37, and 0.32, respectively^32,33^. The ‘E’ values are obtained from the force curves (Nanoscope analysis), for the Ag NPs and Ag_2_Al NPs the values are 83.2 GPa and 109.7 GPa, respectively. The estimated shear stress (τ_max_) values of the Ag NPs and Ag_2_Al NPs are 0.089 MPa and 0.2 MPa, respectively; the alloy Ag_2_Al NPs adhesion is more than twice the pure Ag NPs adhesion on the silicon surface. Further, the work function of Ag_2_Al alloy NPs are also investigated by the KPFM (Kelvin Probe Force Microscopy) and compared with the pure Ag NPs, related to this discussion is presented in the supplementary information (Fig. S1).

### Surface Morphology of Ag_2_Al alloy nanoparticles

**Figure 4:**
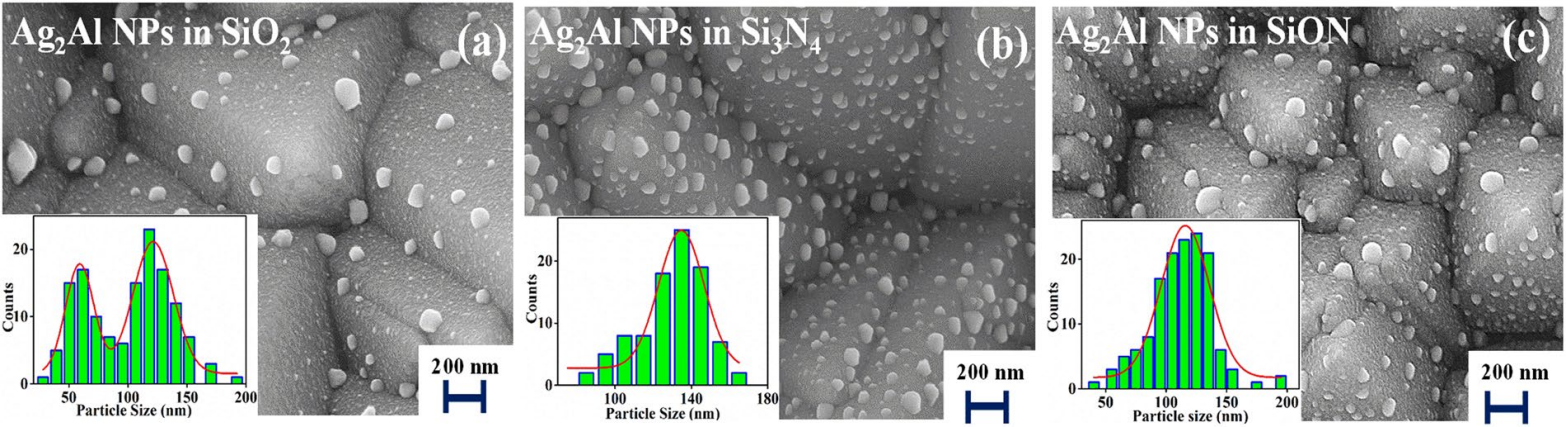
SEM micrographs of the Ag_2_Al NPs embedded in (**a**) ~70 nm SiO_2_, (**b**) ~50 nm Si_3_N_4_ and (**c**) ~60 nm SiON thin films matrix on the textured Si wafer. Inset of the each SEM micrograph shows the NPs’ size histogram.

Figure 4a–c show SEM micrographs of the Ag_2_Al NPs (surface morphology and NPs’ size distribution) embedded in 70 nm SiO_2_, 50 nm Si_3_N_4_, and 60 nm SiON thin films matrix on the textured Si wafers. The alloy NPs embedded in the SiO_2_ film (Fig. 4a) has shown average particles size distribution of ~60 and ~120 nm (for the NPs’ average size estimation the area weighted mean diameter is used). The NPs in the Si_3_N_4_ film has shown average size of ~135 nm with the more surface coverage (Fig. 4b). Whereas the alloy NPs embedded in the SiON film has shown average size of ~115 nm (Fig. 4c), the size distribution is in Gaussian shape that suggests the minimum size deviation^3^.

The NPs formation by the solid-state dewetting process has driven by the substrate and metal film surface energies difference, which breaks the metal film into nanoislands^34^. In this process; the supporting substrate/film surface roughness and metal film’s thermal conductivity are important parameters, which can affect the diffusion and merger of small metal clusters at growth sites during the NPs formation. The measured surface roughness is nearly same (~0.34 ± 0.11 nm) for all three dielectric films, so, which is not going to play any critical role in the NPs formation. Thus, the thermal conductivity of the dielectric film can play a crucial role in the variation of NPs growth; the minimum thermal conductivity of SiO_2_ (1.3 W/m-K) led to two different NPs’ size distribution, whereas the maximum value of Si_3_N_4_ (20 W/m-K) led to the single size distribution^35^. Whereas the SiON film thermal conductivity is ~12 W/m-K^36^, this intermediate value led to the more uniform Ag_2_Al alloy NPs with sizes distribution between 90 to 140 nm.

### Optical Properties

**Figure 5:**
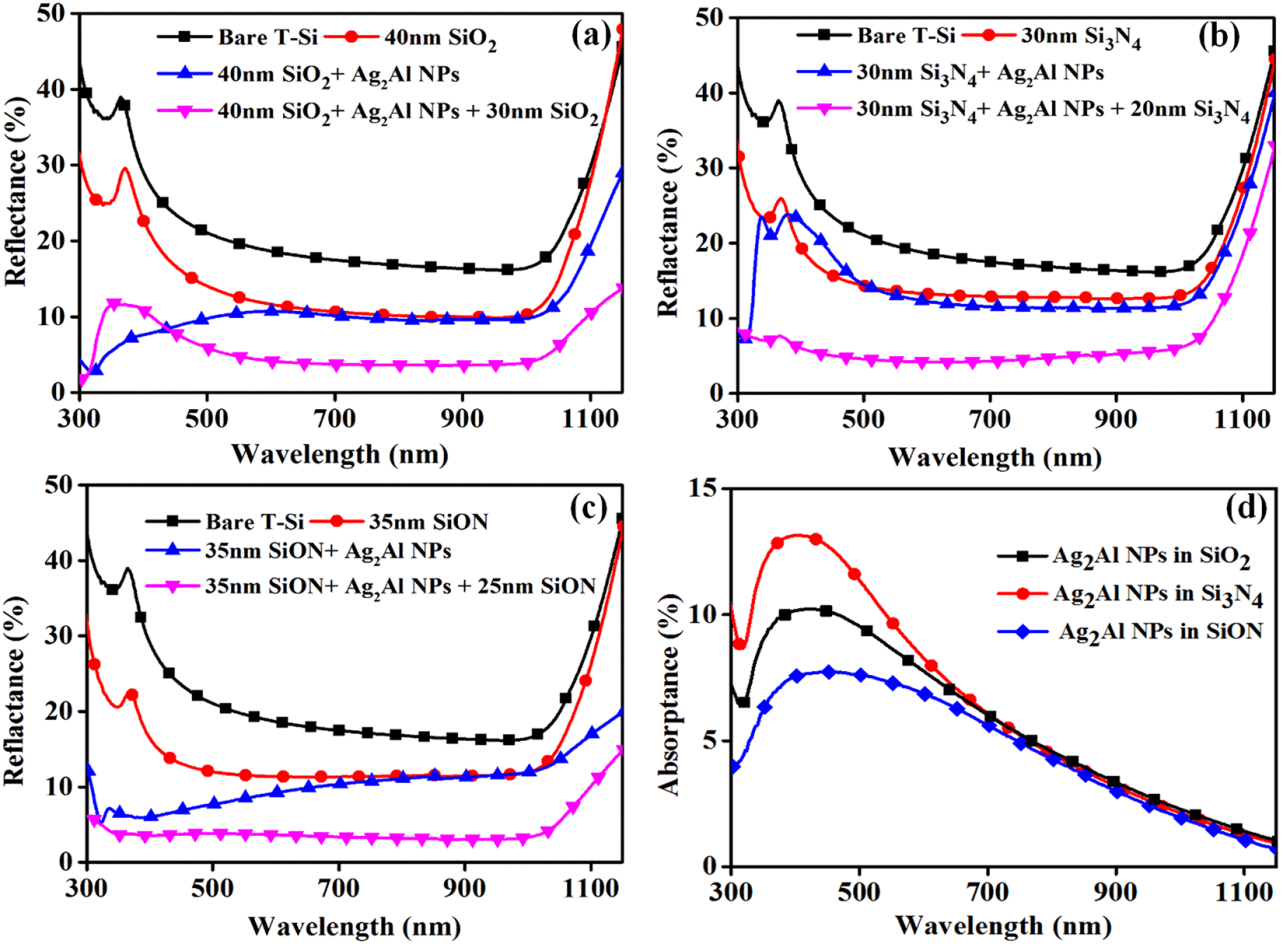
Total reflectance (diffuse + specular) spectra of Ag_2_Al NPs embedded in (**a**) 70 nm SiO_2_, (**b**) 50 nm Si_3_N_4_, (**c**) 60 nm SiON dielectric thin films deposited on textured silicon wafers, (**d**) Absorptance spectra of Ag_2_Al NPs embedded in same films deposited on glass substrates.

Figure 5a–c show total reflectance from the T-Si surface after integrating plasmonic hybrid structure (Ag_2_Al NPs embedded in SiO_2_, Si_3_N_4,_ and SiON thin film matrices) in the wavelength range from 300 to 1150 nm. The average light reflectance from the bare T-Si wafer is ~22.7%, which is considered as a reference. To select a suitable dielectric matrix thickness for the alloy metal NPs integration on a T-Si surface for maximum reflectance reduction, the quarter-wavelength law of destructive interference (near NPs’ LSPR region of 400 to 410 nm) is adopted^37^. The refractive indices of SiO_2_, Si_3_N_4_ and SiON are 1.48, 1.97 and 1.7, respectively. From the quarter-wavelength law (t = λ/4n; Where ‘t’ is the thickness of the dielectric layer, ‘λ’ is the wavelength of incident light, and ‘n’ is the refractive index of the dielectric layer), the SiO_2_, Si_3_N_4_ and SiON layers thicknesses are ~70 nm, ~50 nm and ~60 nm, respectively. First, with ~40 nm SiO_2_, ~30 nm Si_3_N_4_ and ~35 nm SiON spacer layer thicknesses, the average reflectance from the Si wafer reduced to ~14.5%. After the Ag_2_Al NPs integration on 40 nm SiO_2_, 30 nm Si_3_N_4_ and 35 nm SiON spacer layers reflectance reduced to 10.5%, 13.8%, and 9.2%, respectively. Finally, with 30 nm SiO_2_, 20 nm Si_3_N_4_ and 25 nm SiON capping layers on the Ag_2_Al NPs, the reflectance further reduced to ~7.2%, ~6.8%, and ~3.6%, respectively. From the above observations, the average layer thickness of ~60 nm of any dielectric layer still can provide the light trapping. However, one needs to consider the effect of spacer layer thickness for different dielectric matrices, which is very sensitive to the metal NPs’ surface plasmon mode induced near-fields coupling into the Si^5^. Further, reflectance spectra of hybrid plasmonic structures normalized to a reference bare T-Si surface are also presented in the supplementary information (Fig. S2).

The Ag_2_Al alloy NPs on the SiO_2_ and SiON spacer layers led to the reflectance reduction in the surface plasmon resonance region. Whereas, with the Si_3_N_4_ spacer layer, slight increase in reflectance in resonance region and a small decrease in reflectance in the off-resonance region (Fig. 5b). The spacer layer can reduce the refractive index gradient between the silicon and air, which can break the symmetry of polarization of the NPs that can lead to the preferential incoupling of the plasmon-induced field with more intensity into the Si wafer. But, the spacer layer also influences the growth of NPs (Fig. 4), which can affect the light interaction by two ways; (i) the small size NPs can increase the parasitic absorption, and (ii) the destructive interference (hybridization of LSPR modes) due to large coverage/size of the NPs.

After the capping layers integration, the Ag_2_Al alloy NPs led to the reflectance reduction in both surface plasmon resonance and off-resonance region of the NPs. The light-NPs interaction at the nanoscale is very sensitive to the physical environment of NPs besides NPs’ size and shape. A capping layer can provide homogeneous dielectric environment around the NPs, which can enhance the alloy NPs’ near-field intensity by minimizing the SPRs dephasing losses^20^. The reflectance reduction in the entire polychromatic spectral region is due to the effective NPs’ near fields coupling with the sandwich structure instead of just the spacer layer, which is led by the broad angle light forward scattering in all wavelengths^4,19^. In the case of large size NPs, the capping layer is also required for reducing phase mismatch between the incident and scattered light fields (back scattering due to the Fano resonance losses) in the LSPR region (Fig. 5b), which further improves the light forward scattering^22^. The hybrid plasmonic structure of 35 nm SiON/Ag_2_Al NPs/25 nm SiON provided the high density of optical modes, which led to an increase in absorption in the silicon wafer with the minimum average reflectance of ~3.6% in 300 to 1150 nm wavelength region (Fig. 5c)^19^.

One should also know parasitic absorptance losses by the metal NPs, to estimate the effective absorption efficiency of the silicon wafer for the photo-conversion. Figure 5d shows absorptance of the plasmonic structure (on the glass) comprised of alloy NPs embedded in the dielectric medium. The absorptance is estimated using the relation^3^; A = 100 − T_tot_ − R_tot_, where T_tot_ and R_tot_ are the total transmittance and total reflectance of alloy NPs deposited on a glass substrate, respectively. Average absorptance of the Ag_2_Al NPs embedded in the SiO_2_, Si_3_N_4_, and SiON films is ~5.8%, ~6.8% and ~4.6%, respectively, in the spectral region of 300 to 1150 nm. In the LSPR region, the absorptance is maximum due to the parasitic absorption by the NPs. The minimum loss with the SiON film can be either based on the extinction of dielectric material itself or slight variation in the size/density of the NPs. To confirm the plasmonic effect and the superiority of alloy NPs over pure metal NPs, theoretical modeling is required. The elementary theoretical analysis using a finite-element method of alloy NPs’ scattered light into the silicon wafer, and angular/spatial distribution of NPs’ far-fields at the Si/Ag_2_Al NP interface is presented in the supplementary material.

### Electrical Properties

**Figure 6:**
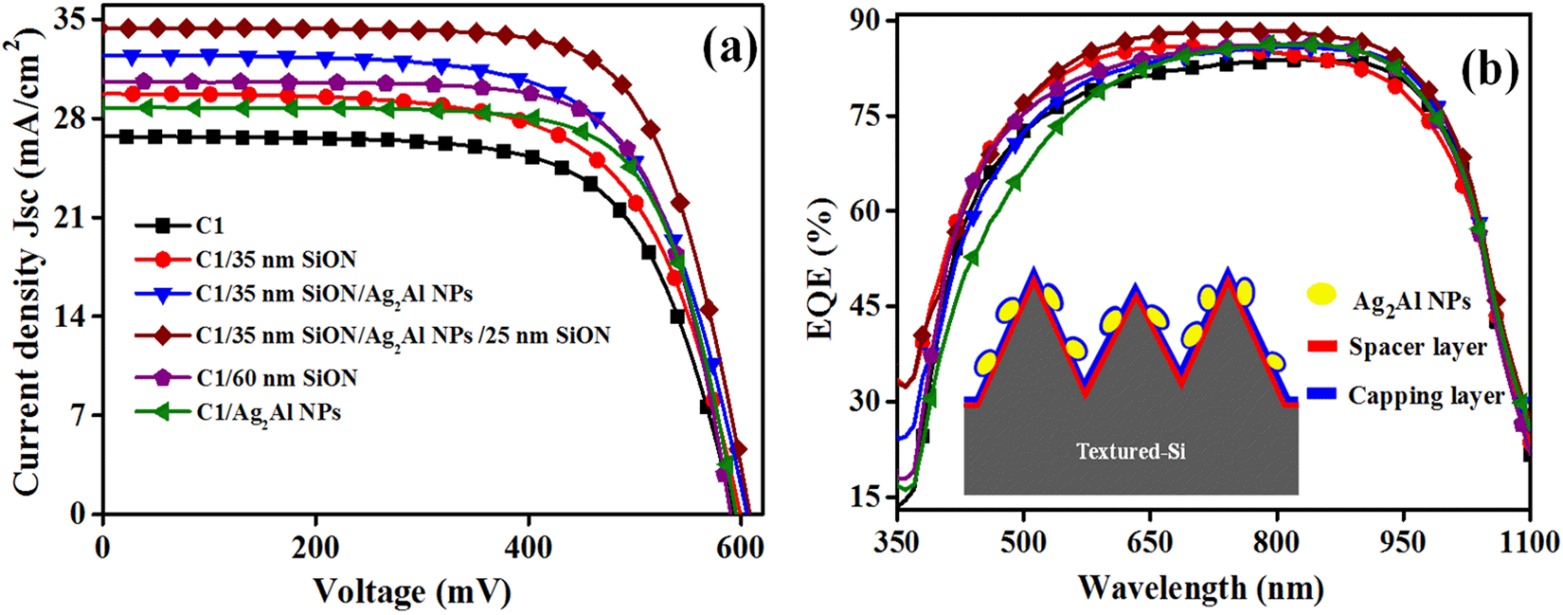
(**a**) J-V graphs, and (**b**) EQE spectra of the bare cell (C1), C1/35 nm of SiON spacer layer, C1/35 nm of SiON/Ag_2_Al NPs, C1/35 nm of SiON/Ag_2_Al NPs/25 nm SiON capping layer, C1/60 nm SiON, and C1/Ag_2_Al NPs. Inset of the EQE spectra shows schematic of PSSC.

To investigate the photo-conversion, we integrated the hybrid plasmonic structure on the conventional textured Si solar cells. Figure 6a shows the J-V graphs of bare Si solar cell (C1), C1 with ~60 nm SiON dielectric film only, C1 with Ag_2_Al NPs only, and C1 with optimized hybrid plasmonic structure (35 nm SiON/Ag_2_Al NPs/25 nm SiON); the corresponding photovoltaic parameters are also summarized in Table 1. The statistical variation of the cells’ short-circuit current (J_sc_), open circuit voltage (V_oc_), Fill Factor (FF), and power conversion efficiency (η) are also presented in the supplementary information (Fig. S3). The bare cell C1 has shown J_sc_ of 26.27 mA-cm^−2^, V_oc_ of 594.76 mV, FF of 69.40, and η of 10.82%. With the plasmonic hybrid structure, the J_sc_ (from 26.27 to 34.61 mA-cm^−2^) and η (from 10.82% to 15.04%) of the cell enhanced impressively in comparison to the bare cell C1.

**Table 1:** Photovoltaic parameters of solar cells; bare cell (C1), C1/35 nm of SiON spacer layer, C1/35 nm of SiON/Ag_2_Al NPs, C1/35 nm of SiON/Ag_2_Al NPs/25 nm SiON capping layer, C1/60 nm SiON, and C1/Ag_2_Al NPs.

Device	Jsc (mA/cm^2^)	Voc (mV)	FF	η (%)
Bare Cell (C1)	26.27	594.76	69.40	10.82
C1/35 nm SiON	29.84	595.20	71.59	12.81
C1/35 nm SiON/Ag_2_Al NPs	33.72	599.41	72.51	14.58
C1/35 nm SiON/Ag_2_Al NPs/25 nm SiON	34.61	601.03	72.44	15.04
C1/60 nm SiON	30.57	596.43	72.17	13.15
C1/Ag_2_Al NPs	28.74	598.18	72.05	12.38

There is a consistent J_sc_ enhancement from cells with different configurations in comparison to the bare cell C1; (a) C1/35 nm SiON is 13.58% (from 26.27 to 29.84 mA-cm^−2^), (b) C1/60 nm SiON is 16.37% (from 26.27 to 30.57 mA-cm^−2^), (c) C1/35 nm SiON/Ag_2_Al NPs is 28.36% (from 26.27 to 33.72 mA-cm^−2^), (d) C1/Ag_2_Al NPs is 9.4% (from 26.27 to 28.74 mA-cm^−2^), and (e) C1/35 nm SiON/Ag_2_Al NPs/25 nm SiON is 31.74% (from 26.27 to 34.61 mA-cm^−2^). The enhancement in photocurrent of the cell is due to the plasmonic effects from the NPs and antireflection effects from thin dielectric films. The better performance of PSSCs primarily by the alloy NPs’ near fields coupling via spacer/capping layers, and further light effective scattering into the silicon wafer^19,20^. However, the enhancement of η (39.0%) is larger than the enhancement of J_sc_ (31.74%), which is due to the improvement in V_oc_ (from 594.76 to 601.03 mV) and FF (from 69.40 to 72.44). The modification in V_oc_ and FF is primarily due to the improvement in surface passivation by the spacer layer that can reduce the series resistance of the plasmonic device^38^.

The quantum efficiency analysis can also provide the better understanding of optical-to-electrical conversion efficiency in surface plasmon resonance and off-resonance regions of the NPs that cumulatively influence the J_sc_ of PSSCs (Fig. 6a). The EQE spectra of all cells are presented in Fig. 6b, which elucidate the incident photon to current conversion efficiency modification as a function of the wavelength for the bare cell (C1), C1/35 nm of SiON spacer layer, C1/35 nm of SiON/Ag_2_Al NPs, C1/35 nm of SiON/Ag_2_Al NPs/25 nm SiON capping layer, C1/60 nm SiON, and C1/Ag_2_Al NPs. With 35 nm SiON spacer layer, the photocurrent of the device improved mainly in 300–500 nm wavelength region due to an anti-reflection effect. After the Ag_2_Al NPs integration on 35 nm SiON film, a small reduction in photocurrent observed near the LSPR region (~410 nm) despite reduced reflectance (Fig. 5c), which is due to parasitic absorption by the NPs (Fig. 5d). The metal nanostructures absorb the incident light along with the light scattering, the absorption/scattering is very sensitive to the metal nanoparticles’ morphology. The parasitic absorption of light by the NPs will not contribute to the carrier generation near the LSPR region (Fig. 6b). Usually, the parasitic absorption is very small in large size (>100 nm) metal NPs, we also have observed a very small parasitic absorption by the alloy NPs (Fig. 5d). However, the device photocurrent in the off-resonance region (600–1000 nm) of the NPs enhanced in comparison to the bare cell, which is due to the preferential light forward scattering by the alloy NPs’ dipole-dipole and/or dipole-quadrupole fields.

With the 25 nm SiON capping layer, the net positive effect in photocurrent enhancement is observed in the polychromatic spectral region (300 to 1100 nm wavelength region). Fahim *et al*. also reported the textured multi-crystalline solar cell photocurrent enhancement with 60 nm Au NPs embedded in SiN_x_ matrix, which attributed to the enhancement in light forward scattering and near-field by the NPs^19^. With 60 nm thin SiON layer alone, the EQE of the cell is improved due to the standard antireflection effect, but smaller than the hybrid plasmonic structure. The cell C1 with the Ag_2_Al NPs only has shown less improvement in the J_sc_ due to unfavourable light forward scatting from the NPs due to the large dielectric environment variation, and can also to some extent charge carrier trap states at the Si/NPs interface. The quantum efficiency analysis also proved the hybrid plasmonic layer role in an increase in light transmission through the silicon wafer by an enhancement in the photocurrent of the silicon solar cell.

## Conclusions

The Ag_2_Al alloy NPs (Ag-10 wt. % Al) are embedded in the dielectric matrix for minimizing the optical losses from a silicon wafer. The light interaction with the 35 nm SiON/Ag_2_Al NPs/25 nm SiON hybrid plasmonic structure reduced the reflectance of the textured silicon wafer from 22.7% to ~3.6% along with minimal parasitic absorption loss in the wavelength region 300 to 1150 nm. Reduced reflectance with the hybrid plasmonic structure led to the overall photocurrent improvement of the plasmonic cell is from 26.27 to 34.61 mA-cm^−2^ and cell efficiency from 10.82% to 15.04% in comparison to bare silicon cell. This work demonstrated the hybrid plasmonic structure role by combining the dielectric thin film anti-reflection effects and alloy NPs’ high density of optical modes for the light confinement, to reduce the net optical loss in the textured silicon wafer and further on the silicon solar cell performance.

## Experimental Methods

### Fabrication of alloy NPs in dielectric matrix

The Ag-10 wt. % Al alloy NPs, and dielectric films of different thicknesses are deposited on p-type (100) oriented textured silicon (T-Si) of 180 ± 20 µm thickness and also on glass substrates by Radio Frequency (RF) magnetron sputtering system. The system working pressure was ~1.3 × 10^−2^ mbar for all depositions. Before the deposition, the glass substrates cleaned ultrasonically in de-ionize water, acetone, and isopropyl alcohol sequentially; the T-Si wafers cleaned by the standard Radio Corporation of America method followed by a native oxide removal in 5% hydrofluoric acid. The Ag-10 wt. % Al thin films of mass thickness 12 ± 0.2 nm co-sputtered, and subsequently annealed for 1 h at 350 °C in the N_2_ ambient to form self-assembled NP arrays. The alloy film deposition calibrated to have desired chemical composition with 30 W and 10 W RF power for Ag and Al metals, respectively. To embed the alloy NPs in a dielectric matrix, first dielectric spacer layer sputtered on the T-Si wafer, afterwards, the Ag_2_Al NPs prepared on the spacer layer, and again, the same dielectric film deposited on the alloy NPs as a capping layer. Silicon dioxide (SiO_2_), silicon nitride (Si_3_N_4_), and silicon oxynitride (SiON) films used as dielectric matrices (thickness details of the spacer and capping layers provided in Table 2). The SiO_2_ and Si_3_N_4_ films deposited with 120 W and 75 W RF power, respectively; for the SiON films deposition by co-sputtered with the same RF power applied for the SiO_2_ and Si_3_N_4_ targets.

**Table 2:** Thickness of spacer and capping layers in which the Ag_2_Al NPs embedded.

Sr. No.	Dielectric material	Spacer layer thickness (nm)	Capping layer thickness (nm)
1.	SiO_2_	40	30
2.	Si_3_N_4_	30	20
3.	SiON	35	25

### Device Fabrication

Optimized hybrid plasmonic layer (35 nm SiON/Ag_2_Al alloy NPs/25 nm SiON) is integrated on the conventional crystalline textured Si solar cells. For a cell fabrication, p-type Czochralski wafer (resistivity of 1–5 Ω-cm) used as a base, a shallow ~0.3 µm n-type layer has grown on the wafer by POCl_3_ diffusion at ~880 °C for 40 min. Silver and aluminum materials are screen printed on the front and rear sides of a p-n junction, respectively; followed by co-firing for 5 s at ~800 °C to form the Ohmic contacts on both sides of the p-n junction. The standard Si_3_N_4_ anti-reflection coating (ARC) step avoided, to investigate the hybrid plasmonic layer effect on the cell performance.

### Characterizations

Energy Dispersive X-ray spectroscopy (EDX) analysis carried out for the alloy composition estimation. The structural and phase analysis of Ag-Al alloy NPs conducted by X-Ray Diffraction (XRD; Rigaku Ultima IV) technique with the Cu Kα source (*λ*_CuKα_ = 1.5406 Å). The optical constants of Ag-Al alloy NPs estimated by a variable angle spectroscopic ellipsometer (VASE, J. A. Woollam M-2000) in 300–1100 nm wavelength region. The chemical and oxidation states of Ag and Al in the Ag_2_Al NPs determined by X-ray Photoelectron Spectroscopy (XPS) using the Mg K*α* (1253.6 eV) source. For nanoindentation, the AFM (Bruker, Dimension Icon) employed to estimate the elastic modulus (from shear stress analysis), and work function analysis by KPFM of the alloy NPs. Surface topology of self-assembled Ag_2_Al NP arrays analyzed by Scanning Electron Microscope (SEM, Carl Zeiss EVO-50). Optical measurements performed by Perkin-Elmer Lambda 1050 UV-Vis-NIR spectrophotometer with an attachment of 150 mm integrating sphere in 300–1100 nm wavelength region. The plasmonic solar cells’ current density-voltage (*J-V*) characteristics recorded using Class AAA solar simulator (Oriel Sol3A, Newport, USA). External Quantum Efficiency (EQE) spectra recorded using quantum efficiency measurement system (SpeQuest, ReRa Solutions, The Netherlands) with AM 1.5 G incident solar spectrum.

## Electronic supplementary material

Supplementary Information

## References

[1] Atwater HA, Polman A (2010). Plasmonics for improved photovoltaic devices. Nat. Mater..

[2] Thouti E, Sharma AK, Sardana SK, Komarala VK (2014). Internal quantum efficiency analysis of plasmonic textured silicon solar cells: surface plasmon resonance and off-resonance effects. J. Phys. D: Appl. Phys..

[3] Morawiec S (2013). Self-assembled silver nanoparticles for plasmon-enhanced solar back reflectors: correlation between structural and optical properties. Nanotechnology.

[4] Sardana SK, Komarala VK (2016). Optical properties of hybrid plasmonic structure on silicon using transparent conducting-silver nanoparticles-silicon dioxide layers: the role of conducting oxide layer thickness in antireflection. J. Opt..

[5] Parashar PK, Sharma RP, Komarala VK (2017). Double-layer antireflection from silver nanoparticle integrated SiO_2_ layer on silicon wafer: effect of nanoparticle morphology and SiO_2_ film thickness. J. Phys. D: Appl. Phys..

[6] Gerard D, Gray SK (2015). Aluminium plasmonics. J Phys. D: Appl. Phys..

[7] Akimov YA, Koh WS (2010). Resonant and nonresonant plasmonic nanoparticles enhancement for thin-film silicon solar cells. Nanotechnology.

[8] Zhang S (2011). Substrate-Induced Fano Resonances of a Plasmonic Nanocube: A Route to Increased-Sensitivity Localized Surface Plasmon Resonance Sensors Revealed. Nano Lett..

[9] Lin DS (1971). The adhesion of metal films to glass and magnesium oxide in tangential shear. J. Phys. D: Appl. Phys..

[10] Parashar PK, Sharma RP, Komarala VK (2016). Plasmonic silicon solar cell comprised of aluminum nanoparticles: Effect of nanoparticles’ self-limiting native oxide shell on optical and electrical properties. J. Appl. Phys..

[11] Knight MW (2013). Aluminum for plasmonics. ACS Nano.

[12] Langhammer C, Schwind M, Kasemo B, Zoric I (2008). Localized surface plasmon resonances in aluminum nanodisks. Nano Lett..

[13] Zhang Y (2013). Improved multicrystalline Si solar cells by light trapping from Al nanoparticle enhanced antireflection coating Opt. Mater. Exp..

[14] Lei ZW (2012). Morphology and optical absorption change of Ag/SiO_2_ core-shell nanoparticles under thermal annealing. Appl. Phys. Lett..

[15] Barron LW, Neidrich J, Kurinec SK (2007). Optical, electrical and structural properties of sputtered aluminum alloy thin films with copper, titanium and chromium additions. Thin Solid Films.

[16] Prieto P (2012). XPS study of silver, nickel and bimetallic silver-nickel nanoparticles prepared by seed mediated growth. Appl. Surf. Sci..

[17] Yang G, Fu XJ, Sun JB, Zhou J (2013). Optical properties of aluminum-silver alloy films deposited by magnetron sputtering. J. Alloy. Compd..

[18] Song T, Gao Y, Zhang Z, Zhai Q (2011). Dealloying behavior of rapidly solidified Al-Ag alloys to prepare nanoporous Ag in inorganic and organic acidic media. Cryst. Eng. Comm..

[19] Fahim NF (2012). Enhanced photocurrent in crystalline silicon solar cells by hybrid plasmonic antireflection coatings. Appl. Phys. Lett..

[20] Ho WJ, Lee YY, Lin CH, Yeh CW (2015). Performance enhancement of plasmonics silicon solar cells using Al_2_O_3_/In NPs/TiO_2_ antireflective surface coating. Appl. Surf. Sci..

[21] McIntosh KR, Johnson LP (2009). Recombination at textured silicon surfaces passivated with silicon dioxide. J. Appl. Phys..

[22] Powell AW, Smith JM (2016). Mediating Fano losses in plasmonic scatterers by tuning the dielectric environment. Appl. Phys. Lett..

[23] Pandey RK, Patil LS, Bange JP, Gautam DK (2004). Growth and characterization of silicon nitride films for optoelectronics applications. Opt. Mater..

[24] Konofaos N (2004). Dielectric properties of CVD grown SiON thin films on Si for MOS microelectronic devices. Semicond. Sci. Technol..

[25] Fuggle JC, Kallne E, Watson LM, Fabian DJ (1977). Electronic structure of aluminum-noble metal alloys studied by soft-X-ray photoelectron spectroscopies. Phys. Rev. B.

[26] Thouti E, Kumar S, Komarala VK (2016). Enhancement of minority carrier lifetimes in n- and p-type silicon wafers using silver nanoparticle layers. J Phys. D: Appl. Phys..

[27] Johansoson G (1973). Calibration of electron spectra. *J. Electron*. Spectroscopy and related phenomenon.

[28] Sritharan T, Li YB, Xu C, Zhang S (2008). Oxidation of Al-Au intermetallics and its consequences studied by x-ray photoelectron spectroscopy. J. Mater. Res..

[29] Hoflund GB, Hazos ZF, Salaita GN (2000). Surface characterization study of Ag, AgO, and Ag2O using x-ray photoelectron spectroscopy and electron energy-loss spectroscopy. Phys. Rev. B.

[30] Oliver WC, Pharr GM (2004). Measurement of hardness and elastic modulus by instrumented indentation: Advances in understanding and refinements to methodology. J. Mater. Res..

[31] Rivas LM (2015). Nanomechanical characterization of nanostructured bainitic steel: Peak Force Microscopy and Nanoindentation with AFM. Scientific Reports.

[32] Smithells, C. J. *Metals reference book 5*^th^*edition* pp. 976 (Butterworth & Co. Ltd. London, 1976).

[33] Carvill, J. *Mechanical engineer’s data handbook* pp. 237 (Butterworth-Heinemann London, 1994).

[34] Thompson CV (2012). Solid-state dewetting of thin films. Annu. Rev. Mater. Res..

[35] Tanyeli I (2013). Effect of surface type on structural and optical properties of Ag nanoparticles formed by dewetting. Opt. Express.

[36] Mahan GD, Bartkowiak M (1999). Wiedemann-Franz law at boundaries. Appl. Phys. Lett..

[37] Kim KH, Park QH (2013). Perfect anti-reflection from first principles. Scientific Reports.

[38] Fahim N (2012). Efficiency enhancement of screen-printed multicrystalline silicon solar cells by integrating gold nanoparticles via a dip coating process. Opt. Express.

