# Supplementary material for: Engineered optical properties of silver-aluminum alloy nanoparticles embedded in SiON matrix for maximizing light confinement in plasmonic silicon solar cells
Source: Sci Rep. 2017 Oct 2;7:12520. doi: 10.1038/s41598-017-12826-1 (PMC5624887; doi:10.1038/s41598-017-12826-1)
Supplement: Supplementary file 1 — Supplementary Information [file 41598_2017_12826_MOESM1_ESM.pdf]

Supplementary Information

Engineered optical properties of silver-aluminum alloy nanoparticles embedded in SiON matrix for maximizing light confinement in plasmonic silicon solar cells

Piyush K. Parashar and Vamsi K. Komarala*

Centre for Energy Studies, Indian Institute of Technology Delhi, New Delhi-110016, India

KPFM analysis

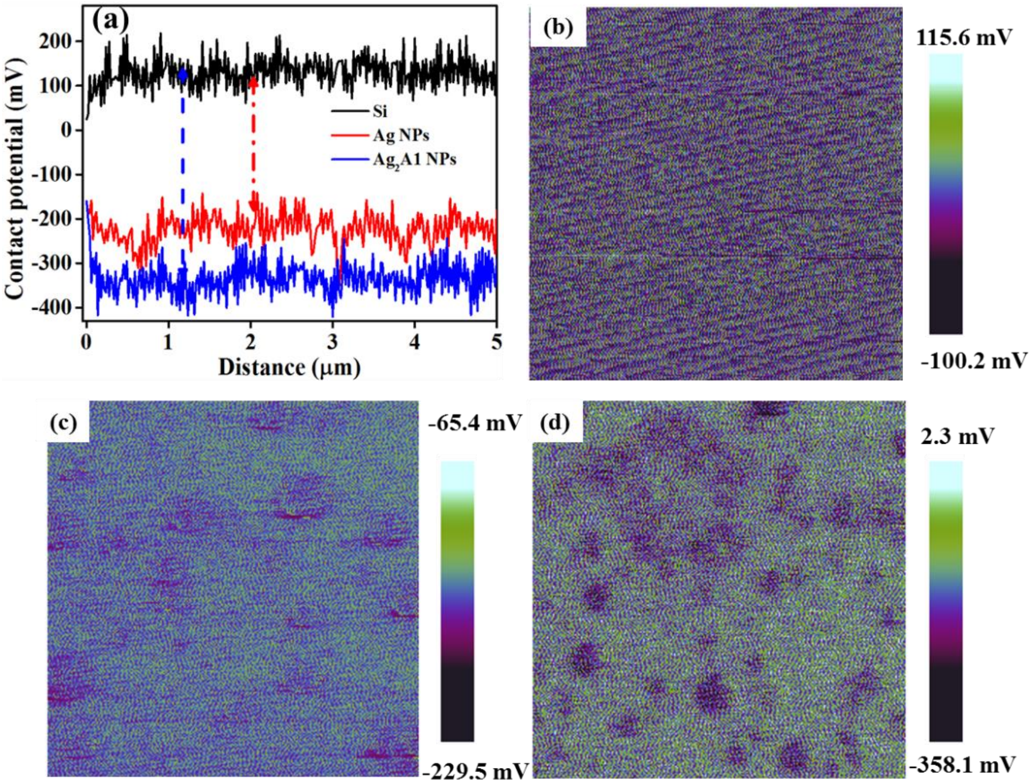

Fig. S1. (a) Section analysis of surface potential profiles obtained from the KPFM surface maps of (b) Si wafer, (c) Ag NPs on the Si wafer, and (d) Ag_2Al NPs on the Si wafer.

The KPFM analysis is used to estimate the contact potential difference (V_{CPD}) between the tip and Ag NPs/Ag_2Al NPs with the following relation[1],

$$V_{CPD} = \frac{\emptyset_{tip} - \emptyset_{sample}}{q} \quad (1)$$

Where; ϕ_{tip} and ϕ_{sample} are the tip and sample work functions, respectively, and q is the Columbic charge. The ϕ_{tip} value of 4.85±0.10 eV is obtained by scanning a freshly cleaved highly oriented pyrolytic graphite sample, whose work function is 4.6 ± 0.1 eV. The V_{CPD} profiles are used to estimate the work functions, which are obtained from the section analysis (Fig. S1a) of the KPFM micrographs presented in Figs. S1b-d related to the Si wafer, Ag NPs, and Ag_2Al NPs. The work functions (ϕ) of bare Si, Ag NPs and Ag_2Al NPs are 4.18±0.1 eV, 4.96±0.1 eV, and 5.13±0.1 eV, respectively. The higher work function of alloy NPs for the same size is due to the Al presence. Usually, the higher metal work function assures the better Ohmic contact at the Si/SiON interface and can play a crucial role in electronic band alignment between the Ag_2Al NPs and Si/SiON interface[2].

Normalised Reflectance

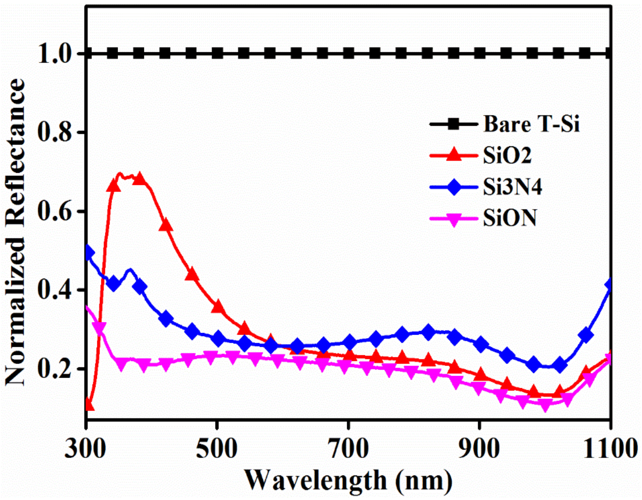

Fig. S2 (a) Normalized reflectance of hybrid plasmonic structures comprised of the Ag_2Al NPs embedded in 70 nm SiO_2, 50 nm $Si_3 N_4$, and 60 nm SiON dielectric films.

The normalized reflectance spectra of Ag_2Al NPs embedded in 70 nm SiO_2, 50 nm Si_3N_4 and 60 nm SiON to the bare T-Si reflectance have shown in Fig. S2. The reflectance is reduced substantially for all cases in comparison to the bare T-Si reflectance. The Ag_2Al NPs embedded in 60 nm SiON film exhibited minimum reflectance in 300-1100 nm spectral region due to the reduced Fano resonance effect, and light forward scattering due to favoured resonant dipole-dipole/dipole-quadrupole fields interaction of the Ag_2Al NPs in the homogeneous dielectric environment.

Statistical variation of J-V characteristics

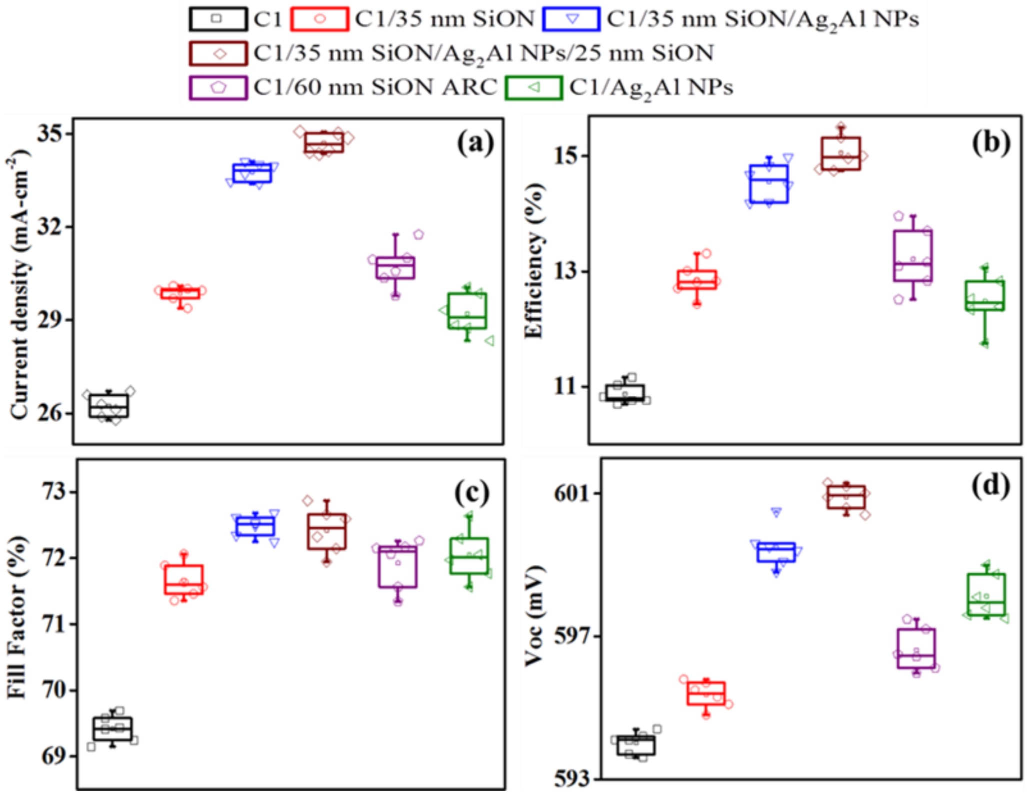

Fig. S3. Statistical variation of (a) J_{sc}, (b) V_{oc}, (c) FF and (d) η of cells; bare cell (C1), C1/35 nm of SiON spacer layer, C1/35 nm of SiON/Ag_2Al NPs, C1/35 nm of SiON/Ag_2Al NPs/25 nm SiON capping layer, C1/60 nm SiON, and C1/ Ag_2Al NPs.

Theoretical background

The morphology dependent metal NPs' surface plasmon field distribution is very important aspect to understand the preferential light forward scattering into any substrate for maximum light confinement. A theoretical study is also performed for the elementary interpretation of experimental observations. The modelling is accomplished using the Finite Element Method (FEM) solver (COMSOL Multiphysics v4.3b). For these calculations, Periodic Boundary Condition (PBC) is used in x- and y- directions to simulate the NPs array. Perfectly Matched Layer (PML) boundary conditions are used in the z-direction, which acts as an absorber for the scattered field to prevent any nonphysical scattering from the boundaries. Usually, the self-assembled dewetting method of NPs leads to the formation of oblate shape (height of NPs < diameter of NPs), here we consider the oblate shape NP size with an aspect ratio of 0.6 for the simulations. The oblate shape NPs provide the better broad spectral light trapping than the spherical NPs due to their shape anisotropy, which is advantageous for photovoltaic application.

Figure 1a shows the peak radiative power (scattering efficiency fraction) towards the Si substrate as a function of Ag, Al, and Ag_2Al NPs' size, which is also known as an Albedo [$F_{sub} = \int_{\lambda} \left(\mathrm{Q}_{\mathrm{sca}} / \mathrm{Q}_{\mathrm{ext}} \right)$], where; Q_{sca} and Q_{ext} are the scattering and extinction efficiencies of the NP, respectively. One can see that the fraction of scattering efficiency enhances with an increase of NP size and then stabilizes. The important observation from the Fig. 1a is that the scattering efficiency of the Ag NP is higher than the Al NP due to the favourable dielectric constants (Fig. 2). The Al inclusion (10 %) in the Ag NP increased saturation threshold value of the NP's size without affecting the maximum scattering efficiency fraction.

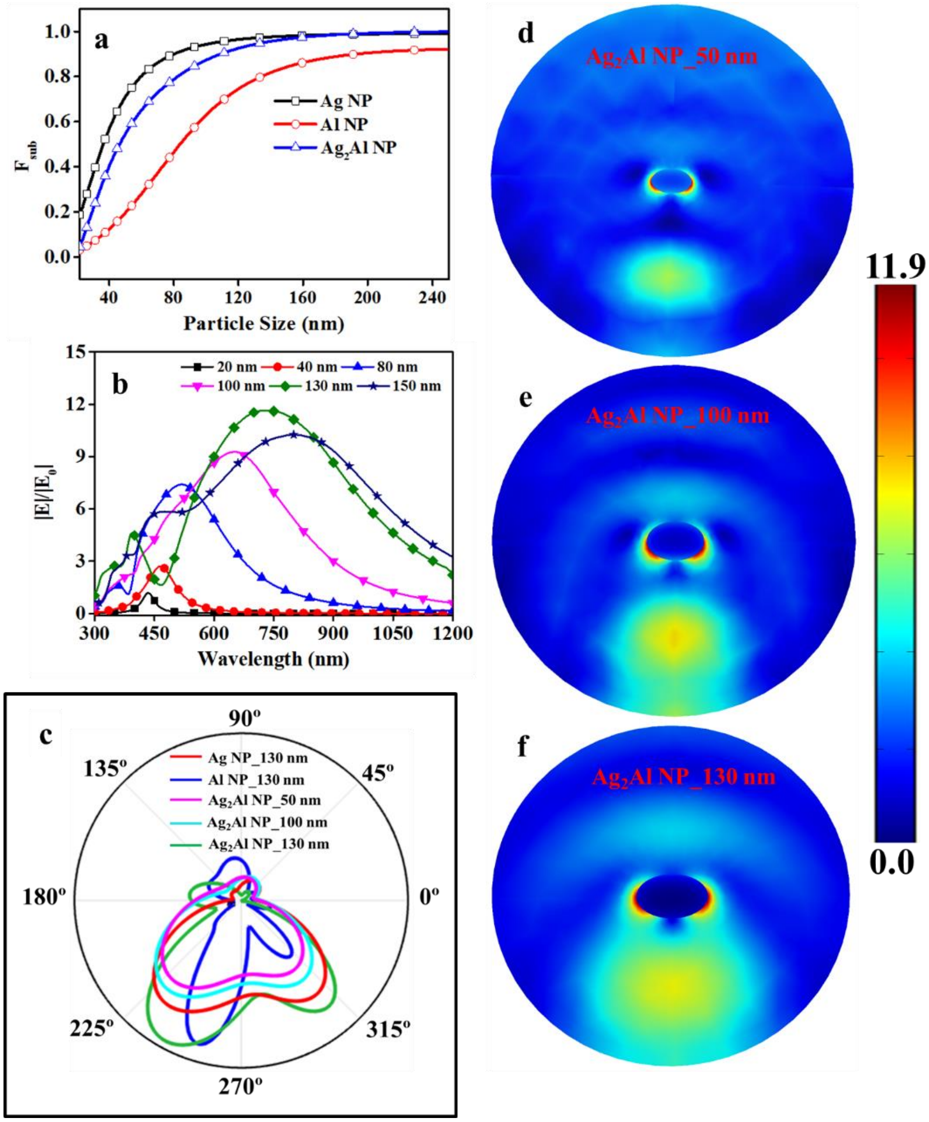

Fig. 1: (a) Fraction of the scattered light (F_{sub}) into the Si substrate as a function of Ag, Al and Ag_2Al (10% Al) NPs' size, (b) Size-dependent normalised electric field of an Ag_2Al NP, (c) 2D far-fields angular distribution of 50 nm, 100 nm and 130 nm Ag_2Al NPs, and also 130 nm Ag NP, 130 nm Al NP, and (d), (e) and (f) are spatial electric field distribution of 50 nm, 100 nm and 130 nm Ag_2Al NPs at the Si/Ag_2Al NP interface, respectively.

Fig. 1b shows wavelength dependent normalized electric field (|E|/|E_0|) plots of 20 to 150 nm Ag_2Al NPs on the Si substrate, where |E| is induced electric field of the NP after light interaction and |E_0| is an incident light electric field. The NP is excited with a normal incidence plane wave $\mathbf{E}_{i,x} = E_0$ exp. ($i \cdot k_0 \cdot y$), where $E_0 = 1$ V-m^{-1}. With an increase of NP size from 20 to 130 nm, the excited NP's dipole (L=1) field intensity is enhanced by six fold (from 1.8 to 11.9), then after the peak intensity is reduced (~8.7) for 150 nm size Ag_2Al NP. These calculations also demonstrate that the optimum NP size should be between 100 to 150 nm for the broadband light trapping into the Si substrate, which supports our experimental observations. Figure 1c shows the 2D far-field angular distribution of 50 nm, 100 nm and 130 nm Ag_2Al NPs, and also 130 nm Ag NP, 130 nm Al NP for the comparison. One can see that the broad angular far-field distribution with large depth from 130 nm Ag_2Al NP, which is better than the same size Ag and Al NPs' far-field distribution. Further, the cross-sectional spatial electric field distribution of 50 nm, 100 nm and 130 nm Ag_2Al NPs at the Si/Ag_2Al NP interface is also estimated, which are presented in Figs. 1d, 1e, and 1f, respectively. One can observe an enhancement in the field distribution with an increase in the NP size.

References:

1. Melitz, W., Shen, J., Kummel, A. C. & Lee, S. Kelvin probe force microscopy and its application. *Surf. Sci. Reps.* **66,** 1-27 (2011).
2. Thouti, E., Kumar, S. & Komarala, V. K. Enhancement of minority carrier lifetimes in n- and p-type silicon wafers using silver nanoparticle layers. *J Phys. D: Appl. Phys.* **49,** 015302 (2016).
